# Supplementary material for: Point-of-Care and Rapid Tests for the Etiological Diagnosis of Respiratory Tract Infections in Children: A Systematic Review and Meta-Analysis
Source: Antibiotics (Basel). 2022 Sep 3;11(9):1192. doi: 10.3390/antibiotics11091192 (PMC9494981; doi:10.3390/antibiotics11091192)
Supplement: Supplementary file 1 [file antibiotics-11-01192-s001.zip › Figures S1-S4.pdf]

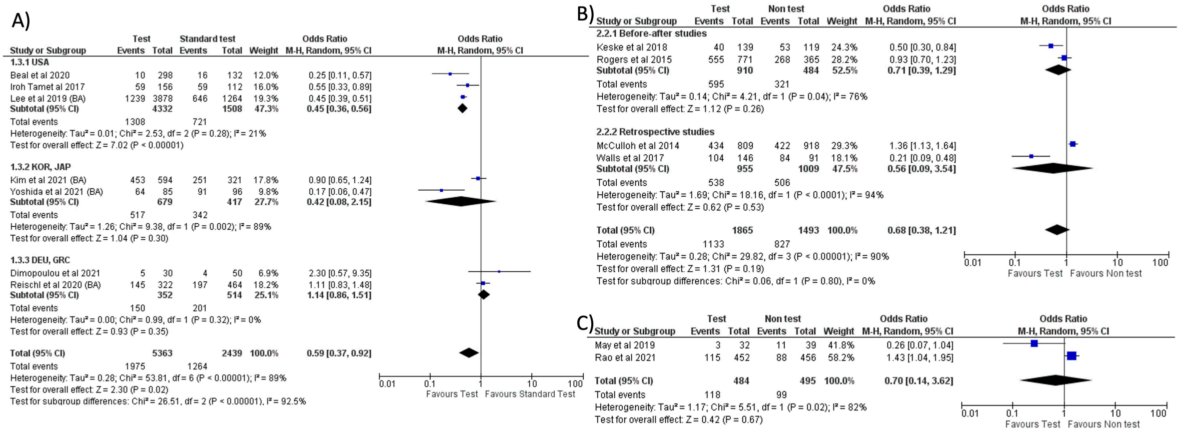

Figure S1. Forest Plot of prescription of antibiotics after implementation of FA-RP: A) FA-RP versus Standard test stratified for different countries; B) FA-RP versus clinical diagnosis in observational studies; C) FA-RP versus clinical diagnosis in randomized controlled trial

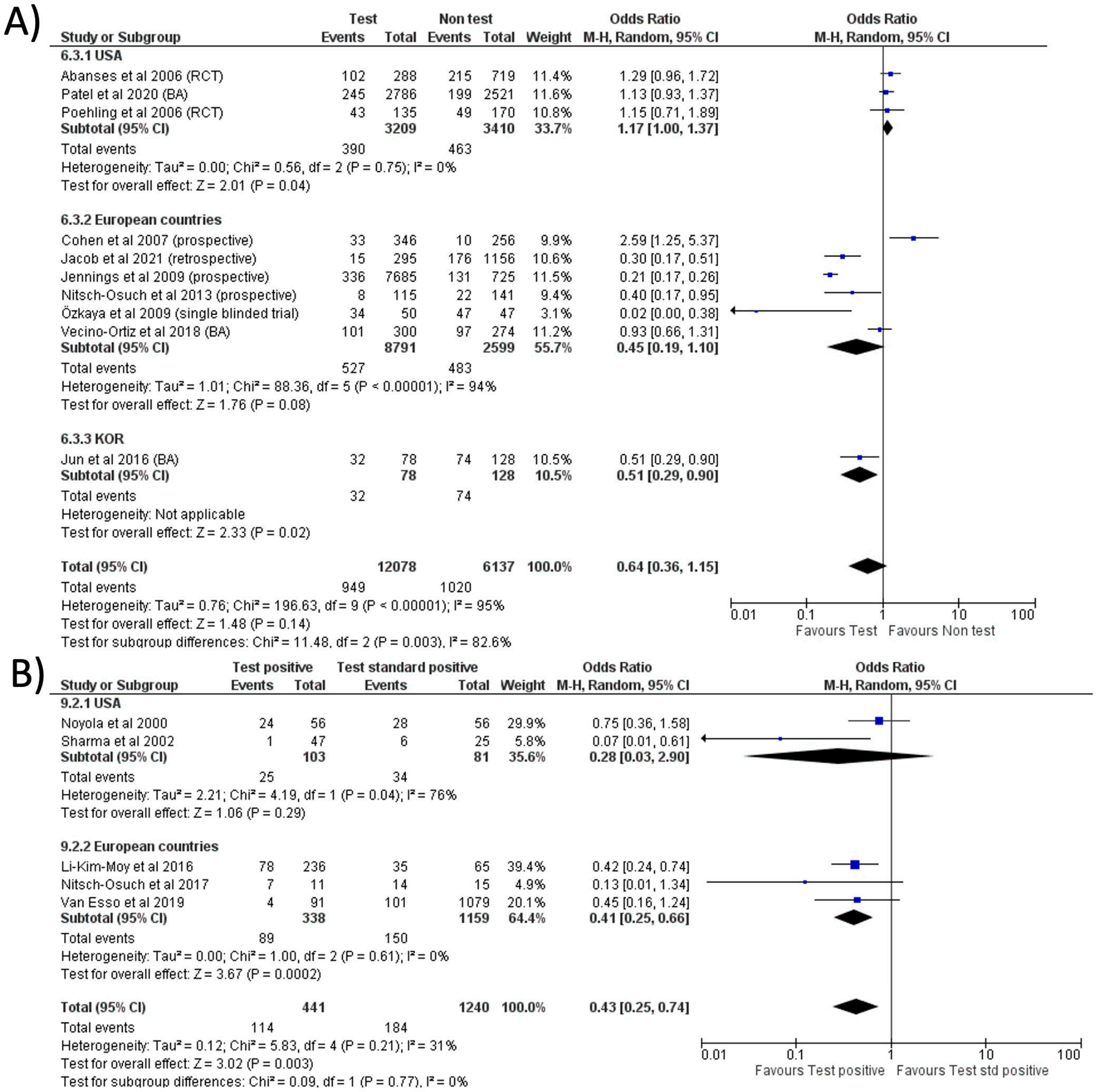

Figure S2. Forest Plot of prescription of antibiotics after implementation of RIDT: A) RIDT vs clinical diagnosis stratified by countries; B) RIDT positive vs standard test positive stratified by countries

A)

Study or Subgroup	Test Events	Test Total	Non test Events	Non test Total	Weight	Odds Ratio M-H, Random, 95% CI
**6.4.1 Before-after studies**						
Jun et al 2016	32	78	74	128	13.5%	0.51 [0.29, 0.90]
Patel et al 2020	245	2786	199	2521	14.6%	1.13 [0.93, 1.37]
Vecino-Ortiz et al 2018	101	300	97	274	14.3%	0.93 [0.66, 1.31]
**Subtotal (95% CI)**		**3164**		**2923**	**42.4%**	**0.88 [0.60, 1.28]**
Total events	378		370			
Heterogeneity: Tau² = 0.07; Chi² = 6.98, df = 2 (P = 0.03); I² = 71%						
Test for overall effect: Z = 0.68 (P = 0.50)						
**6.4.2 Prospective, retrospective studies**						
Cohen et al 2007	33	346	10	256	12.8%	2.59 [1.25, 5.37]
Jacob et al 2021	15	295	176	1156	13.6%	0.30 [0.17, 0.51]
Jennings et al 2009	336	7685	131	725	14.6%	0.21 [0.17, 0.26]
Nitsch-Osuch et al 2013	8	115	22	141	12.2%	0.40 [0.17, 0.95]
**Subtotal (95% CI)**		**8441**		**2278**	**53.2%**	**0.49 [0.17, 1.38]**
Total events	392		339			
Heterogeneity: Tau² = 1.02; Chi² = 46.61, df = 3 (P < 0.00001); I² = 94%						
Test for overall effect: Z = 1.35 (P = 0.18)						
**6.4.3 Cross sectional, single blinded trial**						
Özkaya et al 2009	34	50	47	47	4.4%	0.02 [0.00, 0.38]
**Subtotal (95% CI)**		**50**		**47**	**4.4%**	**0.02 [0.00, 0.38]**
Total events	34		47			
Heterogeneity: Not applicable						
Test for overall effect: Z = 2.63 (P = 0.009)						
**Total (95% CI)**		**11655**		**5248**	**100.0%**	**0.53 [0.26, 1.08]**
Total events	804		756			
Heterogeneity: Tau² = 0.89; Chi² = 166.51, df = 7 (P < 0.00001); I² = 96%						
Test for overall effect: Z = 1.75 (P = 0.08)						
Test for subgroup differences: Chi² = 7.19, df = 2 (P = 0.03), I² = 72.2%						

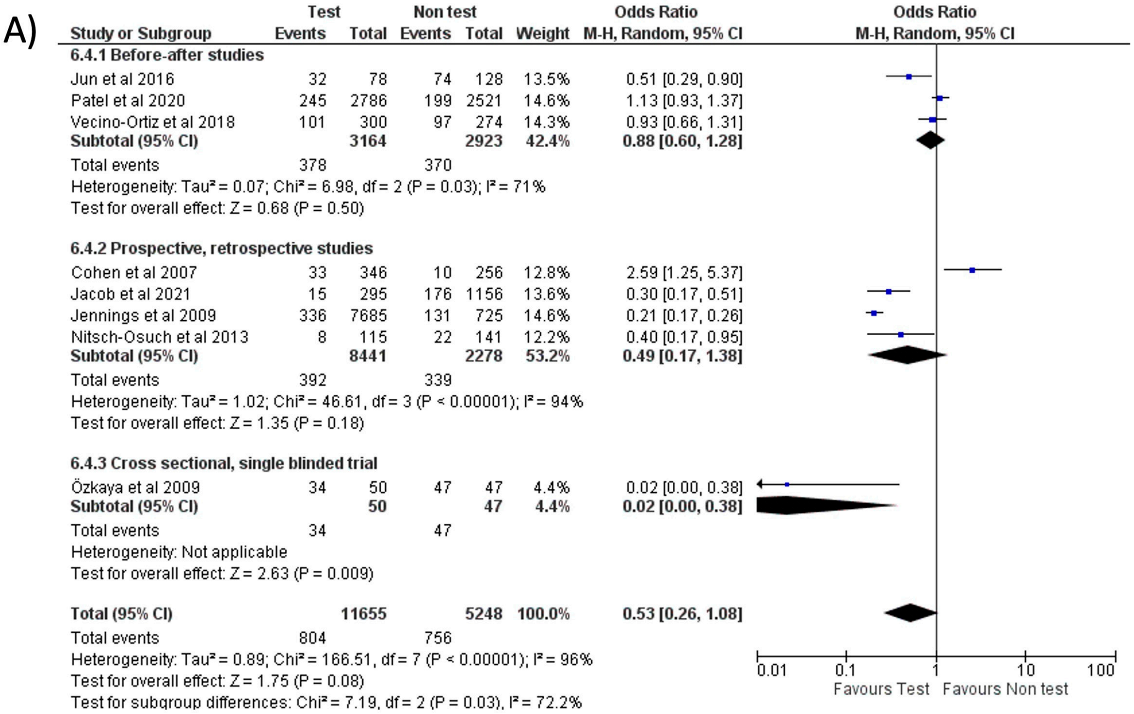

B)

Study or Subgroup	Test Events	Test Total	Non test Events	Non test Total	Weight	Odds Ratio M-H, Random, 95% CI
Abanses et al 2006	102	288	215	719	74.2%	1.29 [0.96, 1.72]
Poehling et al 2006	43	135	49	170	25.8%	1.15 [0.71, 1.89]
**Total (95% CI)**		**423**		**889**	**100.0%**	**1.25 [0.97, 1.60]**
Total events	145		264			
Heterogeneity: Tau² = 0.00; Chi² = 0.14, df = 1 (P = 0.71); I² = 0%						
Test for overall effect: Z = 1.76 (P = 0.08)						

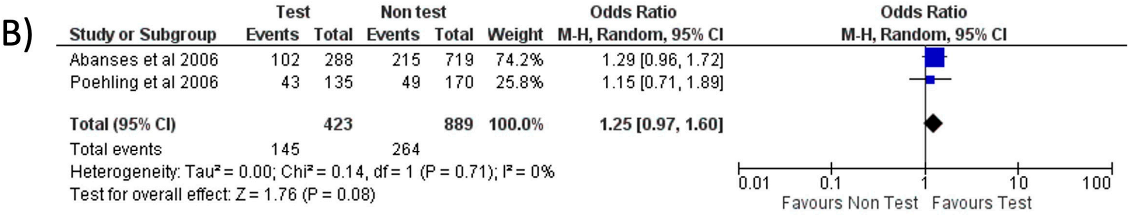

Figure S3. Forest Plot of prescription of antibiotics after implementation of RIDT: A) RIDT vs clinical diagnosis in observational studies; B) RIDT vs clinical diagnosis in RCT

A)

Study or Subgroup	Test Events	Test Total	Standard test Events	Standard test Total	Weight	Odds Ratio M-H, Random, 95% CI
**10.2.1 USA**						
Beal et al 2020	6	29	27	36	20.6%	0.09 [0.03, 0.28]
Echavarría et al 2018	2	113	1	43	12.2%	0.76 [0.07, 8.57]
Lee et al 2019	124	3878	23	1264	25.1%	1.78 [1.14, 2.79]
**Subtotal (95% CI)**		**4020**		**1343**	**57.8%**	**0.49 [0.05, 4.66]**
Total events	132		51			
Heterogeneity: Tau² = 3.40; Chi² = 22.36, df = 2 (P < 0.0001); I² = 91%						
Test for overall effect: Z = 0.62 (P = 0.54)						
**10.2.2 European countries**						
Dimopoulou et al 2021	16	30	33	50	22.3%	0.59 [0.23, 1.49]
Reischl et al 2020	6	322	4	464	19.8%	2.18 [0.61, 7.80]
**Subtotal (95% CI)**		**352**		**514**	**42.2%**	**1.05 [0.29, 3.76]**
Total events	22		37			
Heterogeneity: Tau² = 0.54; Chi² = 2.66, df = 1 (P = 0.10); I² = 62%						
Test for overall effect: Z = 0.08 (P = 0.94)						
**Total (95% CI)**		**4372**		**1857**	**100.0%**	**0.70 [0.22, 2.24]**
Total events	154		88			
Heterogeneity: Tau² = 1.35; Chi² = 25.35, df = 4 (P < 0.0001); I² = 84%						
Test for overall effect: Z = 0.60 (P = 0.55)						
Test for subgroup differences: Chi² = 0.33, df = 1 (P = 0.56), I² = 0%						

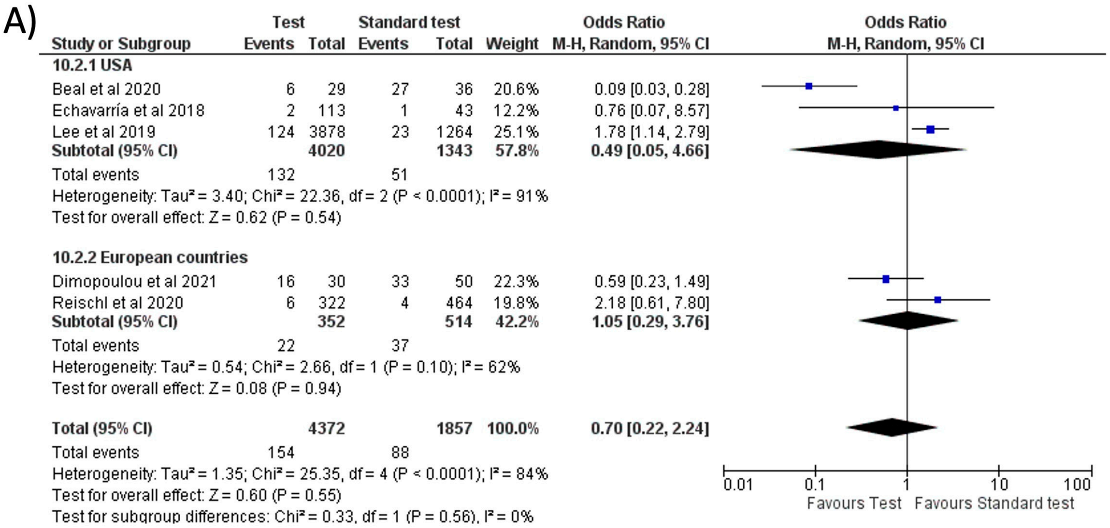

B)

Study or Subgroup	Test Mean	Test SD	Test Total	Standard test Mean	Standard test SD	Standard test Total	Weight	Mean Difference IV, Random, 95% CI
**16.3.1 Before-after studies**								
Kim et al 2021	3.1	4.2	594	3.4	4.3	321	33.9%	-0.30 [-0.88, 0.28]
Kitano et al 2020	8.56	5.13	149	12.82	9.62	1132	32.6%	-4.26 [-5.26, -3.26]
Reischl et al 2020	9.1	5.4	322	8.6	4.4	464	33.5%	0.50 [-0.21, 1.21]
**Subtotal (95% CI)**			**1065**			**1917**	**100.0%**	**-1.32 [-3.70, 1.06]**
Heterogeneity: Tau² = 4.27; Chi² = 61.77, df = 2 (P < 0.00001); I² = 97%								
Test for overall effect: Z = 1.09 (P = 0.28)								

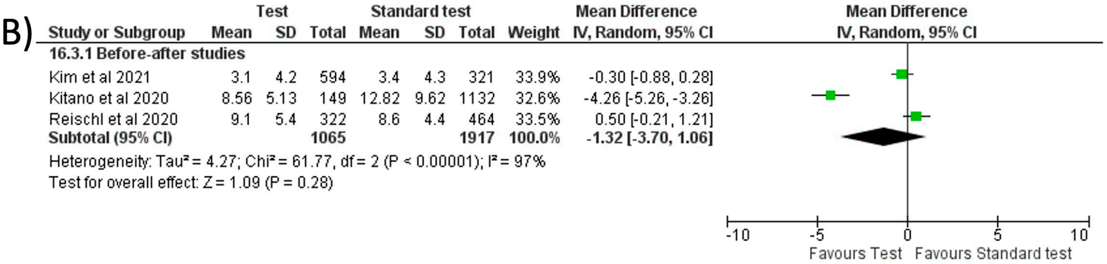

Figure S4. Forest Plot A) of prescription of oseltamivir after implementation of FA-RP versus Standard test stratified for different countries; B) of days of therapy after implementation of FA-RP versus Standard Test
