# Supplementary material for: Point-of-Care and Rapid Tests for the Etiological Diagnosis of Respiratory Tract Infections in Children: A Systematic Review and Meta-Analysis
Source: Antibiotics (Basel). 2022 Sep 3;11(9):1192. doi: 10.3390/antibiotics11091192 (PMC9494981; doi:10.3390/antibiotics11091192)
Supplement: Supplementary file 1 [file antibiotics-11-01192-s001.zip › Supplementary Material File S1.pdf]

Supplementary Material File S1. Search strategy:

1. (newborn* or neonat* or infan* or toddler* or pre-schooler* or preschooler* or child* OR children or adolescen* or pediatr* or paediatr* or youth* or teen or teens or teenage* or kid or kids or baby or babies).mp. [mp=title, abstract, original title, name of substance word, subject heading word, floating sub-heading word, keyword heading word, protocol supplementary concept word, rare disease supplementary concept word, unique identifier, synonyms]

2. Point-of-care ADJ4 Test*.mp. [mp=title, abstract, original title, name of substance word, subject heading word, floating sub-heading word, keyword heading word, protocol supplementary concept word, rare disease supplementary concept word, unique identifier, synonyms]

3. Rapid ADJ4 test*.mp. [mp=title, abstract, original title, name of substance word, subject heading word, floating sub-heading word, keyword heading word, protocol supplementary concept word, rare disease supplementary concept word, unique identifier, synonyms]

4. Near patient test*.mp. [mp=title, abstract, original title, name of substance word, subject heading word, floating sub-heading word, keyword heading word, protocol supplementary concept word, rare disease supplementary concept word, unique identifier, synonyms]

5. Film array.mp [mp=title, abstract, original title, name of substance word, subject heading word, floating sub-heading word, keyword heading word, protocol supplementary concept word, rare disease supplementary concept word, unique identifier, synonyms]

6. Multiplex ADJ2 PCR.mp [mp=title, abstract, original title, name of substance word, subject heading word, floating sub-heading word, keyword heading word, protocol supplementary concept word, rare disease supplementary concept word, unique identifier, synonyms]

7. Multiplex ADJ2 Polymerase Chain Reaction.mp [mp=title, abstract, original title, name of substance word, subject heading word, floating sub-heading word, keyword heading word,

protocol supplementary concept word, rare disease supplementary concept word, unique identifier, synonyms]

8. 2 or 3 or 4 or 5 or 6 or 7

9. exp "Outcome Assessment (Health Care)"/ or outcome*.mp. [mp=title, abstract, original title, name of substance word, subject heading word, floating sub-heading word, keyword heading word, protocol supplementary concept word, rare disease supplementary concept word, unique identifier, synonyms]

10. exp "Costs and Cost Analysis"/ or cost*.ti,ab,kw.

11. exp "Patient Care"/ or patient care*.mp [mp=title, abstract, original title, name of substance word, subject heading word, floating sub-heading word, keyword heading word, protocol supplementary concept word, rare disease supplementary concept word, unique identifier, synonyms]

12. Mortality/ or mortality.fs. or (mortalit* or death* or fatal*).ti,ab,kw. or Morbidity/ or morbidit*.ti,ab,kw.

13. (anti-bacterial* or antibacterial* or anti-viral* or antiviral* or antibiotic* or anti-infective or bactericid* or bacteriocid* or antimicrobial).mp. [mp=title, abstract, original title, name of substance word, subject heading word, floating sub-heading word, keyword heading word, protocol supplementary concept word, rare disease supplementary concept word, unique identifier, synonyms]

14. 9 or 10 or 11 or 12 or 13

15. 1 and 8 and 14

16. 15 not case reports.pt.

17. 16 not (exp HIV Infections/ or exp HIV/ or (HIV or human immunodeficiency virus).mp. or exp Plasmodium/ or plasmodium.mp. or exp Malaria/ or malaria.mp. or exp

Mycobacterium infections/ or mycobacterium infection*.mp or exp Mycobacterium/ or mycobacterium.mp) [mp=title, abstract, original title, name of substance word, subject heading word, floating sub-heading word, keyword heading word, protocol supplementary concept word, rare

18. Limit 17 to yr= "2000 - 2021"
