# Supplementary material for: Point-of-Care and Rapid Tests for the Etiological Diagnosis of Respiratory Tract Infections in Children: A Systematic Review and Meta-Analysis
Source: Antibiotics (Basel). 2022 Sep 3;11(9):1192. doi: 10.3390/antibiotics11091192 (PMC9494981; doi:10.3390/antibiotics11091192)
Supplement: Supplementary file 1 [file antibiotics-11-01192-s001.zip › Tables S1-S4.pdf]

Table S1. NIH Assessment Tools for Observational Studies.

	Q1	Q2	Q3	Q4	Q5	Q6	Q7	Q8	Q9	Q10	Q11	Q12	Q13	Q14	Overall	QUALITY
**Beal et al, 2020**	1	1	0	1	0	1	1	0	1	0	1	0	0	0	**7**	**FAIR**
**Benito - Fernandez et al, 2006**	1	1	NA	1	0	1	1	0	1	0	1	0	1	0	**8**	**FAIR**
**Busson et al, 2019**	1	1	1	1	0	1	1	NA	1	0	1	0	NA	1	**9**	**FAIR**
**Byington et al, 2002**	1	1	1	0	0	1	1	NA	1	0	1	0	NA	0	**7**	**FAIR**
**Cantais et al, 2019**	1	1	1	1	0	1	1	NA	1	NA	1	0	0	0	**8**	**FAIR**
**Cohen et al, 2007**	1	1	NA	0	0	1	1	NA	1	0	1	0	0	0	**6**	**FAIR**
**de La Rocque et al, 2009**	1	1	1	1	1	1	1	NA	1	1	1	0	1	0	**11**	**GOOD**
**Diallo et al, 2019**	1	1	1	1	0	1	1	NA	1	0	1	0	0	0	**8**	**FAIR**
**Dimopoulou et al, 2020**	1	1	1	0	0	1	1	NA	1	0	1	0	NA	0	**7**	**FAIR**
**Hayashi et al, 2018**	1	1	1	1	0	1	1	NA	1	0	1	0	0	1	**9**	**FAIR**
**Iroh Tamet al, 2017**	1	1	1	1	0	1	1	NA	1	0	1	0	NA	0	**8**	**FAIR**
**Jacob et al, 2020**	1	1	1	1	0	1	1	NA	1	0	1	0	0	0	**8**	**FAIR**
**Jennings et al, 2009**	1	1	1	NA	0	1	1	NA	1	0	1	0	0	0	**7**	**FAIR**
**Li-Kim-Moy et al, 2016**	1	1	1	0	0	1	1	NA	1	0	1	0	0	1	**8**	**FAIR**
**Małecki et al, 2017**	1	1	1	1	0	1	1	NA	1	1	1	0	1	0	**10**	**FAIR**
**Maltezou et al, 2008**	1	1	1	0	0	1	1	NA	1	0	1	0	0	1	**8**	**FAIR**
**McCulloh et al, 2013**	1	1	0	1	0	1	1	NA	1	0	1	0	NA	0	**7**	**FAIR**
**McFall et al, 2017**	1	1	1	1	NA	1	1	1	1	0	1	0	NA	0	**9**	**FAIR**
**Nitsch-Osuch et al, 2013**	1	1	0	0	0	1	1	NA	1	0	1	0	0	0	**6**	**FAIR**
**Özkaya et al, 2009**	1	1	1	1	NA	1	1	NA	1	0	1	0	1	0	**9**	**FAIR**
**Pierron et al, 2008**	1	1	0	1	0	1	1	NA	1	0	1	0	0	0	**7**	**FAIR**
**Rao et al, 2019**	1	1	1	1	0	1	1	NA	1	0	1	0	0	0	**8**	**FAIR**
**Schnell et al, 2017**	1	1	1	1	NA	1	1	NA	1	0	1	0	NA	0	**8**	**FAIR**
**Schulert et al, 2013**	1	1	0	0	0	1	1	NA	1	0	1	0	NA	0	**6**	**FAIR**
**Sharma et al , 2002**	1	1	1	1	0	1	1	NA	1	0	1	0	NA	0	**8**	**FAIR**
**Thibeault et al, 2007**	1	1	1	1	NA	1	1	NA	1	0	1	0	NA	0	**8**	**FAIR**

**Van Esso et al, 2019**	1	1	NA	0	0	1	1	0	1	0	0	0	0	0	**5**	**POOR**
**Vest Schneider et a, 2018**	1	1	1	1	0	1	1	NA	1	0	1	0	1	0	**9**	**FAIR**
**Walls et al, 2016**	1	1	1	NA	NA	1	1	NA	1	0	1	0	NA	0	**7**	**FAIR**

Table S2. NIH Assessment Tools for Before and After studies.

	**Q1**	**Q2**	**Q3**	**Q4**	**Q5**	**Q6**	**Q7**	**Q8**	**Q9**	**Q10**	**Q11**	**Q12**	**Overall**	**QUALITY**
**Ayanruoh et al, 2009**	1	1	1	1	NA	1	1	0	0	1	0	NA	**7**	**FAIR**
**Bird et al, 2021**	1	1	1	NA	NA	1	1	0	0	1	0	NA	**6**	**FAIR**
**Crook et al, 2020**	1	1	1	1	0	1	1	0	NA	1	1	NA	**8**	**FAIR**
**Halverson et al, 2011**	1	1	NA	NA	NA	1	1	0	0	0	0	NA	**4**	**POOR**
**Jun et al, 2016**	1	1	1	1	NA	1	1	0	0	1	0	NA	**7**	**FAIR**
**Keske et al, 2018**	1	1	1	1	NA	1	1	0	NA	1	0	NA	**7**	**FAIR**
**Kim et al, 2021**	1	1	1	1	NA	1	1	0	NA	1	1	NA	**8**	**FAIR**
**Kitano et al, 2019**	1	1	1	0	NA	1	1	0	1	1	0	NA	**7**	**FAIR**
**Kose et al, 2016**	1	1	NA	1	NA	1	1	0	0	1	0	NA	**6**	**FAIR**
**Lee et al, 2020**	1	1	1	1	NA	1	1	0	NA	1	0	NA	**7**	**FAIR**
**Mitchell et al, 2018**	1	1	1	1	NA	1	1	0	NA	1	0	NA	**7**	**FAIR**
**Nitsch-Osuch et al, 2017**	1	1	NA	1	NA	1	1	0	0	1	0	NA	**6**	**FAIR**
**O' Callaghan et al, 2019**	1	1	NA	NA	NA	1	1	0	0	1	0	NA	**5**	**FAIR**
**Patel et al, 2020**	1	1	1	NA	NA	1	1	0	0	1	NA	NA	**6**	**FAIR**
**Reischl et al, 2020**	1	1	1	1	NA	1	1	0	NA	1	0	NA	**7**	**FAIR**
**Rogers et al, 2015**	1	1	1	1	NA	1	1	0	NA	1	0	NA	**7**	**FAIR**
**Subramony et al, 2016**	1	1	1	1	NA	1	1	0	NA	NA	1	NA	**7**	**FAIR**
**Vecino-Ortiz et al, 2018**	1	0	1	NA	NA	1	1	0	0	1	NA	NA	**5**	**FAIR**
**Yoshida et al, 2021**	1	1	1	1	NA	1	1	0	NA	1	0	NA	**7**	**FAIR**

Table S3. NIH Assessment Tools for Randomized Controlled Trial.

	Q1	Q2	Q3	Q4	Q5	Q6	Q7	Q8	Q9	Q10	Q11	Q12	Q13	Q14	Overall	QUALITY
**Abanses et al, 2006**	0	1	1	0	0	1	1	1	1	1	1	0	1	0	**9**	**FAIR**
**Bonner et al, 2003**	1	1	1	NA	NA	1	1	1	1	1	1	NR	1	1	**11**	**GOOD**
**Echavarríaet al, 2018**	1	1	1	1	0	1	NR	NR	1	1	1	1	1	1	**11**	**GOOD**
**Iyer et al, 2006**	1	0	0	0	0	1	1	1	1	1	1	1	1	1	**10**	**FAIR**
**May et al, 2019**	1	1	1	0	0	1	1	1	1	1	1	1	1	1	**12**	**GOOD**
**Poehling et al, 2006**	1	1	1	0	0	1	1	1	1	1	1	1	1	1	**12**	**GOOD**
**Rao et al, 2021**	1	1	1	0	0	1	0	1	1	1	1	1	1	1	**11**	**GOOD**
**Wishaupt et al, 2011**	1	1	0	1	0	1	1	1	1	1	1	1	1	1	**12**	**GOOD**

Table S4. NIH Assessment Tools for Case-Control Trial.

	Q1	Q2	Q3	Q4	Q5	Q6	Q7	Q8	Q9	Q10	Q11	Q12	Overall	QUALITY
**Noyola et al, 2000**	1	1	0	1	1	1	0	1	1	1	0	1	**9**	**GOOD**
